# Lanthanide-Functionalized Hydrophilic Magnetic Hybrid Nanoparticles: Assembly, Magnetic Behaviour, and Photophysical Properties

**DOI:** 10.1186/s11671-016-1497-3

**Published:** 2016-05-31

**Authors:** Shuai Han, Yu Tang, Haijun Guo, Shenjun Qin, Jiang Wu

**Affiliations:** College of Science, Hebei University of Engineering, Handan, 056000 People’s Republic of China; Key Laboratory of Nonferrous Metal Chemistry and Resources Utilization of Gansu Province, State Key Laboratory of Applied Organic Chemistry and College of Chemistry and Chemical Engineering, Lanzhou University, Lanzhou, 730000 People’s Republic of China; Hebei Collaborative Innovation Center of Coal Exploitation, Hebei University of Engineering, Handan, Hebei 056038 People’s Republic of China

**Keywords:** Magnetite, Luminescent europium complex, Polyethyleneimine, Multifunctional

## Abstract

The lanthanide-functionalized multifunctional hybrid nanoparticles combining the superparamagnetic core and the luminescent europium complex were successfully designed and assembled via layer-by-layer strategy in this work. It is noted that the hybrid nanoparticles were modified by a hydrophilic polymer polyethyleneimine (PEI) through hydrogen bonding which bestowed excellent hydrophilicity and biocompatibility on this material. A bright-red luminescence was observed by fluorescence microscopy, revealing that these magnetic-luminescent nanoparticles were both colloidally and chemically stable in PBS solution. Therefore, the nanocomposite with magnetic resonance response and fluorescence probe property is considered to be of great potential in multi-modal bioimaging and diagnostic applications.

**Electronic supplementary material:**

The online version of this article (doi:10.1186/s11671-016-1497-3) contains supplementary material, which is available to authorized users.

## Background

The past decade has witnessed an explosion of interest in combining optically active components with magnetic nanoparticles (NPs) in one entity [1]. This new class of hybrid nanocomposite has been successfully employed for biomedical imaging [2], drug delivery [3], sensing [4], etc. Owing to their superparamagnetic behaviours, Fe_3_O_4_ NPs could be manipulated by external magnetic field which makes it easier to separate from solution in further modifications.

Therefore, fluorescent compounds such as semiconducting quantum dots [5], fluorescent organic dyes [6], and metal complexes [7] were commonly incorporated with Fe_3_O_4_ NPs to achieve multifunctional capabilities. Compared with these fluorochromes, lanthanide-based complexes are of particular attractiveness for their large Stokes shift and sharp line-like emission bands [8]. Moreover, their long luminescence lifetimes, typically in the millisecond range, make lanthanide complexes become the most fascinating and useful candidates as time-gated probes in biological systems for the reason that they can typically diminish the background fluorescence of other organic substances [9]. It can be anticipated that the combination of superparamagnetic Fe_3_O_4_ with a lanthanide-based complex would open up the opportunity to provide potential applications in highly sensitive bio-applications.

Although the progress in combination lanthanides and magnetic NPs within one hybrid nanomaterial has advanced rapidly, there are still some challenges for these novel functional materials. One of the most important challenges is the risk of fluorescence quenched by magnetic cores. Therefore, it is of great importance to make sure that both optically active components and magnetic properties were conveyed without compromising by each other. In our synthetic strategy, SiO_2_ was chosen as the coating spacer between the lumophore and the magnetic NPs which could effectively diminish the fluorescence quenching by magnetic cores and prevent Fe_3_O_4_ NPs from aggregation in the solution. Meanwhile the SiO_2_ shell can be easily surface-functionalized and is more biocompatible for further application in biomedical uses [10]. In order to introduce the luminescent centre, we grafted lanthanide complexes directly on the SiO_2_ shell through the covalent bonding. As a strong interaction, covalent bonding can successfully overcome the leaching of fluorescent compounds and enhance the thermal and chemical stabilities of the hybrid nanomaterial.

Polymers are one of the best candidates in modifying the nanostructures for that they can afford controllable functional groups on the surface of nanomaterials. Polyethyleneimine (PEI) is a kind of cationic polyamine owing to the protonation of primary amines on its macromolecular chains. It can be attracted on the surfaces of different materials by hydrogen bonding and can be further functioned for various applications such as removal of heavy metal ions from blood [11], efficient gene delivery in cells [12], nano-drug delivery systems [13], and so on [14]. PEI coating not only offers opportunities to render the Fe_3_O_4_ NPs with excellent hydrophilicity and biocompatibility but also can overcome the fluorescence disturbed by the environment.

**Fig. 1:**
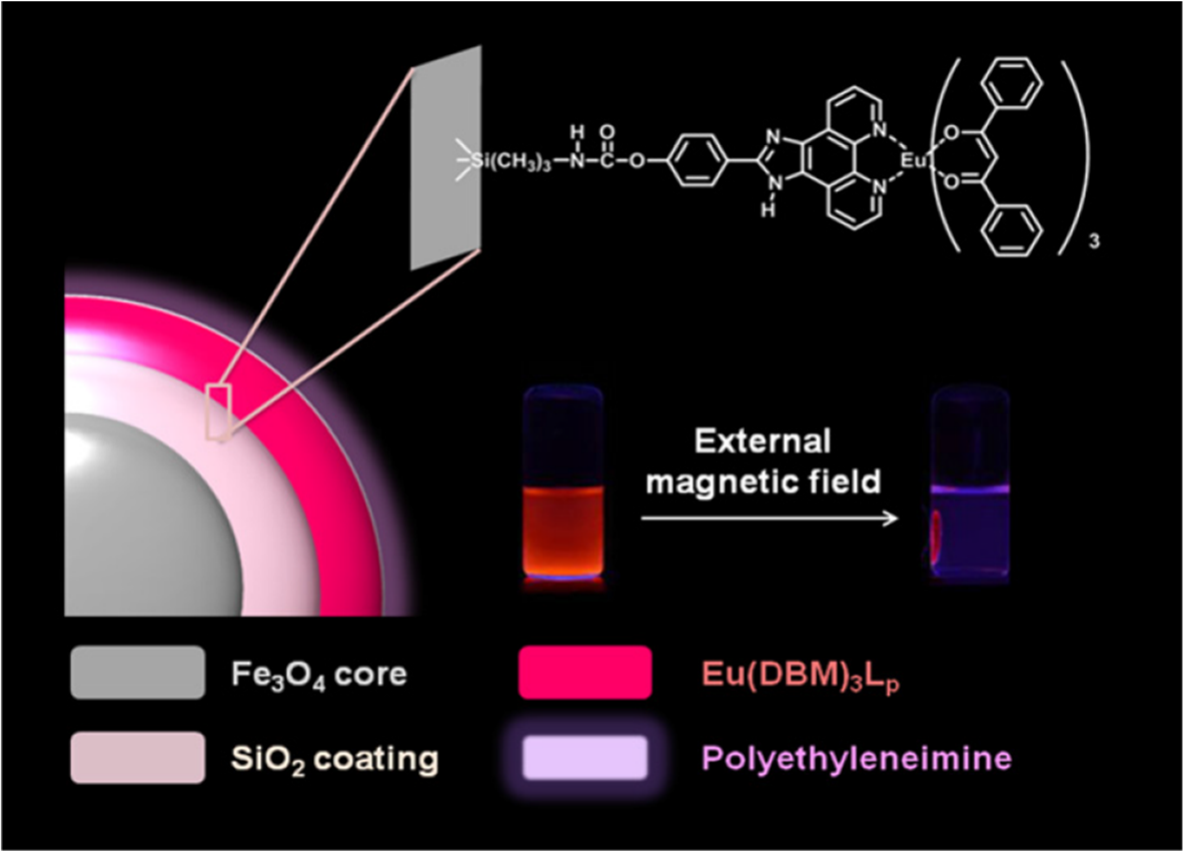
Schematic illustration of fabrication of Fe_3_O_4_@SiO_2_-[Eu (DBM)_3_L_p_]@PEI magnetic-luminescent nanocomposite

Herein, a kind of novel magnetic-luminescent NP has been assembled by the coupling of a europium(III) complex with dibenzoylmethanate (DBM) and 2-(4-hydroxy-phenyl)imidazo[4,5-f]1,10-phenanthroline (L_p_) onto Fe_3_O_4_@SiO_2_ NPs. The obtained NPs which have the imidazo structure and many oxygen and nitrogen atoms on the surface could conjugate with PEI by hydrogen bonding to fabricate a four-component nanocomposite Fe_3_O_4_@SiO_2_-[Eu(DBM)_3_L_p_]@PEI (Fig. 1). Moreover, it is worth mentioning that this nanocomposite could be effectively sensitized by visible light (*λ* > 385 nm), thus to reduce the effect of UV damage on living biological samples [15], making it of great potential in multi-modal biomedical imaging and diagnostic applications.

## Methods

### Materials and Reagents

1,10-Phenanthroline-5,6-dione and ammonium acetate were obtained from Shanxi Xinhua Co. FeCl_3_·6H_2_O, sodium acrylate, and NaOAc were purchased from Tianjin Guangfu Chemical. 3-(Triethoxysilyl)propyl isocyanate was purchased from Jinan Xinna Medicine Science and Technology Co. Other chemicals and solvents were obtained from Aladdin Chemicals Company and were of analytical grade.

### Analysis

NMR spectra were recorded on a Varian Mercury-300B spectrometer. CHN elemental analyses were measured on an Elementar Vario EL analyser; the contents of Eu(III) ions were obtained by inductively coupled plasma-atomic emission spectroscopy (ICP) using an IRIS Advantage ER/S spectrophotometer. The solid-state absorption spectra were recorded with a Shimadzu UV-3100 spectrophotometer. Fourier transform infrared (FTIR) spectra were conducted within the 4000–400 cm^−1^ wave number range using a Nicolet 360 FTIR spectrometer with the KBr pellet technique. Transmission electron microscope (TEM) images were taken on a JEM-100CX II apparatus, and the fluorescence images were taken on an Olympus FluoView 500 laser scanning confocal microscope (FV1000, MPE). Dynamic light-scattering (DLS) measurements were performed at 25 °C using a Malvern Zetasizer Nano ZS. Magnetic measurements were carried out at room temperature with a Lake Shore-735 vibrating sample magnetometer (VSM) magnetometer. The steady-state luminescence spectra and the lifetime measurements were measured on an Edinburgh Instruments FSL920 fluorescence spectrometer, with a 450-W Xe arc lamp as the steady-state excitation source or an Nd-pumped OPOlette laser as the excitation source for lifetime measurements. The solid-state quantum yield was measured using a Model F-3029, Quanta-Phi 6 Integrating Sphere connected with a Horiba Jobin Yvon Fluorolog-3 spectrophotometer.

### Synthesis of the Ligand L_p_

1,10-Phenanthroline-5,6-dione (0.5 g, 2.3 mmol) and ammonium acetate (2.93 g, 66.5 mmol) were dissolved in 5 mL glacial acetic acid. While the mixture was stirred, a solution of 4-hydroxybenzaldehyde (0.283 g, 2.3 mmol) in glacial acetic acid was added dropwise to the mixture. After heating for 3 h, the mixture was then poured in 200 mL water. The solution was neutralized with ammonia to pH = 7 and was then cooled to room temperature. The precipitate was filtered off and washed with large portions of water, then the crude product was purified by recrystallization from a mixture of EtOH and water solution (yield 65 %). *δ*_H_ (400 MHz, DMSO-d_6_) 7.65–7.68 (dd, *J* = 7.6, 4.4 Hz, 1H), 8.37–8.40 (dd, *J* = 7.6 Hz, 1.6 Hz, 1H), 8.98–9.00 (dd, *J* = 4.4 Hz, 1.6 Hz, 1H). Anal. Calcd C, 73.07; H, 3.87; N, 17.94. Found C, 73.37; H, 3.57; N, 17.82.

### Synthesis of Eu(DBM)_3_L_p_

Solid L_p_ (31.2 mg, 0.10 mmol) prepared was mixed with 25 mL of 95 % ethanolic solution of Eu(DBM)_3_(H_2_O)_2_ (86.0 mg, 0.10 mmol, prepared according to the literature), and then the suspension was sonicated for 20 min. After sonication, the reaction system was stirred at room temperature overnight. The yellow precipitate formed was filtered and washed with the mixed solution of ethanol and water ((*v*/*v*) 1:1) and dried in vacuum, to obtain 70.0 mg of Eu(DBM)_3_L_p_. Anal. Calcd C, 67.78; H, 4.01; N, 4.93. Found C, 67.59; H, 4.35; N, 4.85.

### Synthesis of Fe_3_O_4_ NPs

FeCl_3_·6H_2_O (0.54 g), sodium acrylate (1.5 g), and NaOAc (1.5 g) were dissolved in a mixture of 5 mL ethylene and 15 mL diethylene glycol under vigorous stirring. The obtained homogeneous yellow solution was transferred to a Teflon-lined stainless-steel autoclave, sealed, and heated at 200 °C. After reaction for 10 h, the autoclave was cooled to room temperature. The obtained Fe_3_O_4_ NPs were washed several times with ethanol and water and then dried in vacuum for 12 h. The PXRD analysis of the Fe_3_O_4_ NPs is shown in Additional file 1: Figure S1; it could be seen that all the diffraction peaks are indexed to the cubic structure, known for the Fe_3_O_4_ crystal (JCPDS no. 88-0315) and no other peaks are detected, indicating that the products are pure-phase Fe_3_O_4_.

### Synthesis of Fe_3_O_4_@SiO_2_ NPs

The core-shell Fe_3_O_4_@SiO_2_ nanospheres were prepared according to a previously reported method. Typically, the Fe_3_O_4_ NPs was treated with diluted HCl solution by ultrasonication for 10 min. The magnetite NPs were washed and homogeneously dispersed in a mixture of ethanol, deionized water, and concentrated ammonia aqueous solution, followed by the addition of tetraethyl orthosilicate (TEOS; 0.03 g, 0.144 mmol). After being stirred for 6 h, the Fe_3_O_4_@SiO_2_ nanospheres were separated, washed, and then dried in vacuum.

### Synthesis of 2-[4′-{3-(Triethoxysilyl)Propyl}Phenyl]Imidazo[4,5-f]-1,10-Phenanthroline (L_p_-Si)

A batch of 200 mg (7.45 mmol) of L_p_ was homogeneously dispersed in an excess of 3-(triethoxysilyl)propyl isocyanate (2.5 mL) under ultrasonication. Then, the mixture was stirred under argon at 80 °C for 72 h. The mixture was added slowly to 20 mL of cold hexane, and a white-yellow precipitate was formed. The precipitate was filtered off, washed, and then dissolved in ethanol. The solution was filtered, and the ethanol was removed by rotary evaporation. The obtained compound was dissolved in a small portion of dichloromethane (DCM). This DCM solution was added dropwise to 30 mL of cold hexane to reprecipitate the compound. The purified product was filtered off and dried in vacuo. Yield 60 %; *δ*_H_ (400 MHz, DMSO-d_6_): 1.08–1.15 (t, *J* = 17.0 Hz, 9H), 1.51–1.55 (m, 2H), 3.02–3.07 (m, 2H), 3.71–3.76 (q, *J* = 6.8 Hz, 6H), 7.31–7.33 (d, *J* = 8.8 Hz, 2H), 7.79–7.86 (m, 2H), 8.24–8.26 (d, *J* = 7.6 Hz, 2H), 8.91 (dd, *J* = 8.0 Hz, 1.6 Hz, 2H), 9.04 (dd, *J* = 4.4 Hz, 1.6 Hz, 2H), 13.73 (s, 1H).

### Synthesis of Fe_3_O_4_@SiO_2_-L_p_

Two hundred milligrams Fe_3_O_4_@SiO_2_ in 50 mL of dry toluene mixed with 40 mg of L_p_-Si was stirred and refluxed for 8 h. The solid obtained was then separated by centrifugation, washed with ethanol, and dried at room temperature.

### Synthesis of Fe_3_O_4_@SiO_2_–[Eu(DBM)_3_L_p_]

A batch of 200 mg of Fe_3_O_4_@SiO_2_-L_p_ was refluxed with 30 mg of Eu(DBM)_3_(H_2_O)_2_ in ethanol, then the obtained magnetic-luminescent NPs were collected by centrifugation after 24 h, and the excess of unbound complex was thoroughly washed away with ethanol. After drying at 120 °C for 3 h, Fe_3_O_4_@SiO_2_–[Eu(DBM)_3_L_p_] was obtained.

### Synthesis of Fe_3_O_4_@SiO_2_–[Eu(DBM)_3_L_p_]@PEI

A batch of 200 mg of Fe_3_O_4_@SiO_2_-[Eu(DBM)_3_L_p_] was homogeneously dispersed in ethanol under ultrasonication; PEI ethanol solution was then slowly added to the solution. After being fiercely stirred at room temperature for 6 h, the microspheres were separated, washed with ethanol, and then dried in vacuum at 60 °C for 6 h.

## Results and Discussion

### Microstructure Characterization of the Nanocomposite Fe_3_O_4_@SiO_2_-[Eu(DBM)_3_L_p_]@PEI

**Fig. 2:**
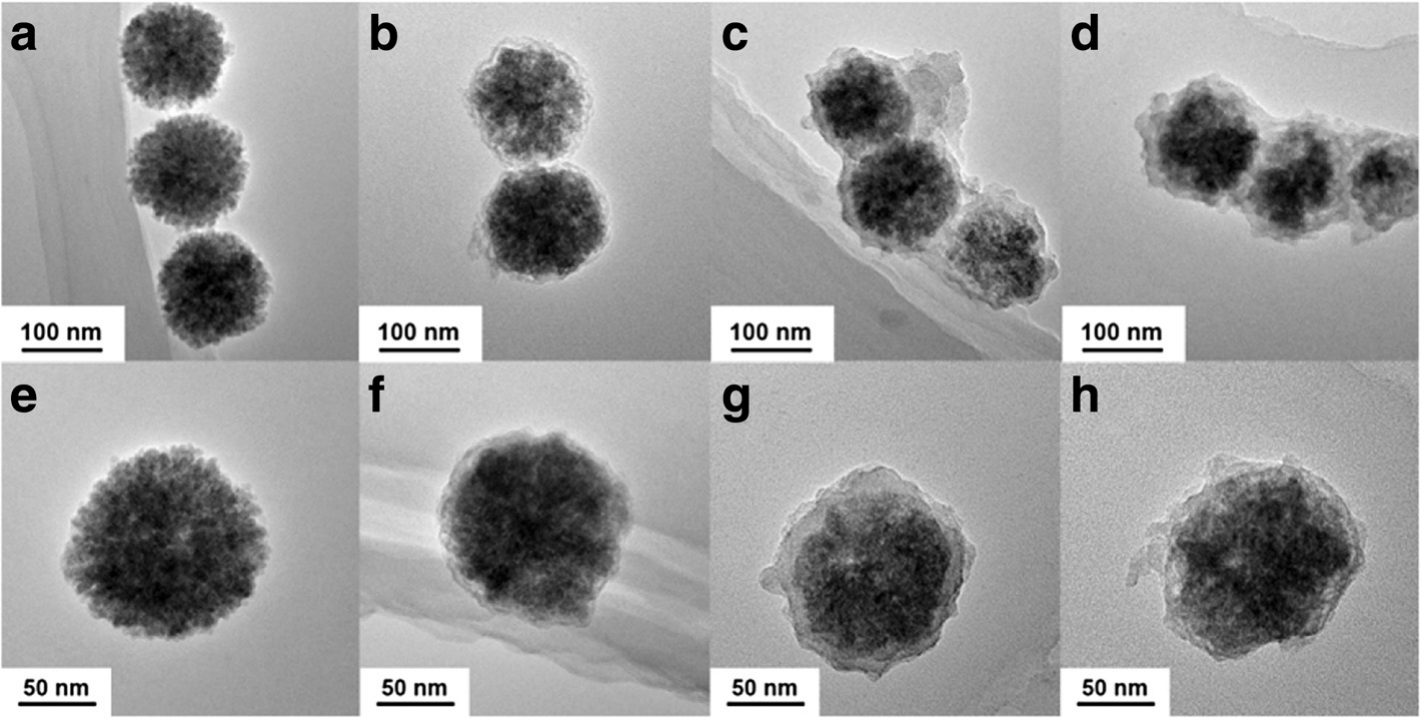
Typical TEM images of **a**, **e** Fe_3_O_4_ NPs; **b**, **f** Fe_3_O_4_@SiO_2_ NPs; **c**, **g** Fe_3_O_4_@SiO_2_-[Eu(DBM)_3_L_p_] NPs; and **d**, **h** Fe_3_O_4_@SiO_2_-[Eu(DBM)_3_L_p_]@PEI NPs

TEM images of the Fe_3_O_4_ magnetic NPs, the Fe_3_O_4_@SiO_2_ composites, the Fe_3_O_4_@SiO_2_-[Eu(DBM)_3_L_p_] magnetic-luminescent dual-functional NPs, and Fe_3_O_4_@SiO_2_-[Eu(DBM)_3_L_p_]@PEI nanocomposite are shown in Fig. 2. It can be observed that the obtained Fe_3_O_4_ NPs are spherical and remarkably uniform with an average size about 100 nm (Fig. 2a, e). Clearly, these NPs are composed of small primary nanocrystals with a size of 6 ~ 8 nm which agrees well with the previous work [16]. TEM images of the resulting Fe_3_O_4_@SiO_2_ composite NPs are shown in Fig. 2b, f. After being coated with a nonporous silica layer, core-shell Fe_3_O_4_@SiO_2_ NPs with a thin silica layer ~10 nm in thickness were obtained. TEM images of the Fe_3_O_4_@SiO_2_-[Eu(DBM)_3_L_p_] NPs which are shown in Fig. 2c, g indicate that the subsequent Eu(III) complex modification process resulted in a continuous and uniform coating on the surface of Fe_3_O_4_@SiO_2_ nanospheres. And TEM images of the PEI-modified nanocomposite are shown in Fig. 2d, h. However, the PEI molecules cannot be seen from the images for the reason that the interference of high-voltage electrons (120 kV, or 200 kV) with the light element compound (C, H, O, N) was too weak to be observed. Besides, the DLS measurements have been performed and the obtained data was shown in Additional file 1: Figure S2. While the mean diameters of the four samples were determined to be 100, 108, 122, and 125 nm which are corresponding with the TEM test results.

The above electron microscope observation results can be further confirmed by zeta potential measurements of the products, which are sensitive mainly to the outer surface of the NPs [17]. The measurements showed that after the SiO_2_ coating, the value zeta potential at physiological pH of 7.4 decreased from −12.7 mV for the starting Fe_3_O_4_ NPs to −28.3 mV. The zeta potential value changed to 9.4 mV upon Eu(III) complex conjugation and increased steeply to 30.2 mV after modifying with PEI, confirming the pronounced increase in the surface amino group density. Thus, the changes in the potential value of the NPs suggested that the surface modifications of SiO_2_, Eu(III) complex and PEI to the NPs were successful.

Furthermore, as a cationic dispersant, PEI could provide both electrostatic repulsion and steric hindrance effect to the nanocomposite and prevent the NPs to coming close enough together to agglomerate into larger NPs. This means that the PEI functionalization on Fe_3_O_4_@SiO_2_-[Eu(DBM)_3_L_p_] could significantly increase the water solubility and stability of the nanocomposite, which was essential for them in biological applications [18].

FTIR spectra were conducted to further verify the obtained products. As seen from Additional file 1: Figure S3, IR spectroscopy provided clear evidence for the layer-by-layer surface modification. Additional file 1: Figure S1b displays the IR spectrum of the bare magnetic NPs, and the characteristic band of Fe_3_O_4_ appeared at about 586 nm. The FTIR spectrum of Fe_3_O_4_@SiO_2_ (Additional file 1: Figure S1c) indicated that the silica shell was coated on the surface of the magnetite cores, which could be confirmed by assignments of the bands Si-O-Si (1085 cm^−1^) and Si-OH (945 cm^−1^), and the characteristic Fe-O peak of Fe_3_O_4_ NPs at 586 cm^−1^ shifted to 589 cm^−1^ in the spectrum of SiO_2_-coated magnetic NPs [19]. Compared with the FTIR spectrum of Fe_3_O_4_@SiO_2_ NPs, the sharp peaks located at 1704 and 1565 cm^−1^ which appeared in the spectrum of Fe_3_O_4_@SiO_2_-[Eu(DBM)_3_L_p_] (Additional file 1: Figure S1d) corresponded to the adsorption of the urea groups (NH-CO-O) and gave strong evidence that isocyanatopropyltriethoxysilane (ICPTES) had been successfully grafted onto Fe_3_O_4_@SiO_2_ NPs [20]. After PEI modification, the peaks for the bending vibration of the N-H group and the stretching vibration of the C-N groups of PEI could be seen at 1570 and 1100 cm^−1^, respectively [21]. All these observations clearly indicated that the modifications were achieved.

**Fig. 3:**
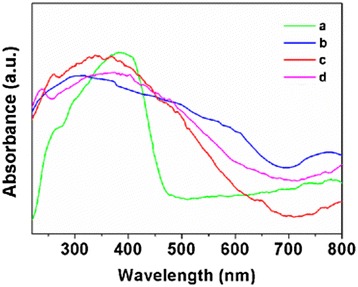
UV-vis absorption spectra of the samples. *a* Eu(DBM)_3_L_p_ complex. *b* Fe_3_O_4_@SiO_2_. *c* Fe_3_O_4_@SiO_2_-[Eu(DBM)_3_L_p_]. *d* Fe_3_O_4_@SiO_2_-[Eu(DBM)_3_L_p_]@PEI

Figure 3 shows UV/vis spectra of the Eu(III) complex, Fe_3_O_4_@SiO_2_ NPs, Fe_3_O_4_@SiO_2_-[Eu(DBM)_3_L_p_] NPs, and Fe_3_O_4_@SiO_2_-[Eu(DBM)_3_L_p_]@PEI nanocomposite in the solid state. The Fe_3_O_4_@SiO_2_-[Eu(DBM)_3_L_p_] NPs showed a wide absorption band at 320–410 nm, which was similar with that of Eu(DBM)_3_L_p_ corresponding to *π*-π* transitions of the ligand. However, a blueshift (15 nm) could be noticed compared to those of Eu(DBM)_3_L_p_, indicating that the complex was grafted onto the matrix. After PEI modification, the functionalized nanocomposite displayed a peak at 230 nm that was typical for PEI. More importantly, comparing with that of Fe_3_O_4_@SiO_2_-[Eu(DBM)_3_L_p_] NPs, the wide absorption band of the Fe_3_O_4_@SiO_2_-[Eu(DBM)_3_L_p_]@PEI nanocomposite exhibited redshift optical absorption (10 nm), which indicated that hydrogen-bonding interaction occurred between the PEI molecules and the surface ligands of the Fe_3_O_4_@SiO_2_-[Eu(DBM)_3_L_p_] nanocomposite [22].

### Magnetic and Photophysical Properties of the Nanocomposite Fe_3_O_4_@SiO_2_-[Eu(DBM)_3_L_p_]@PEI

**Fig. 4:**
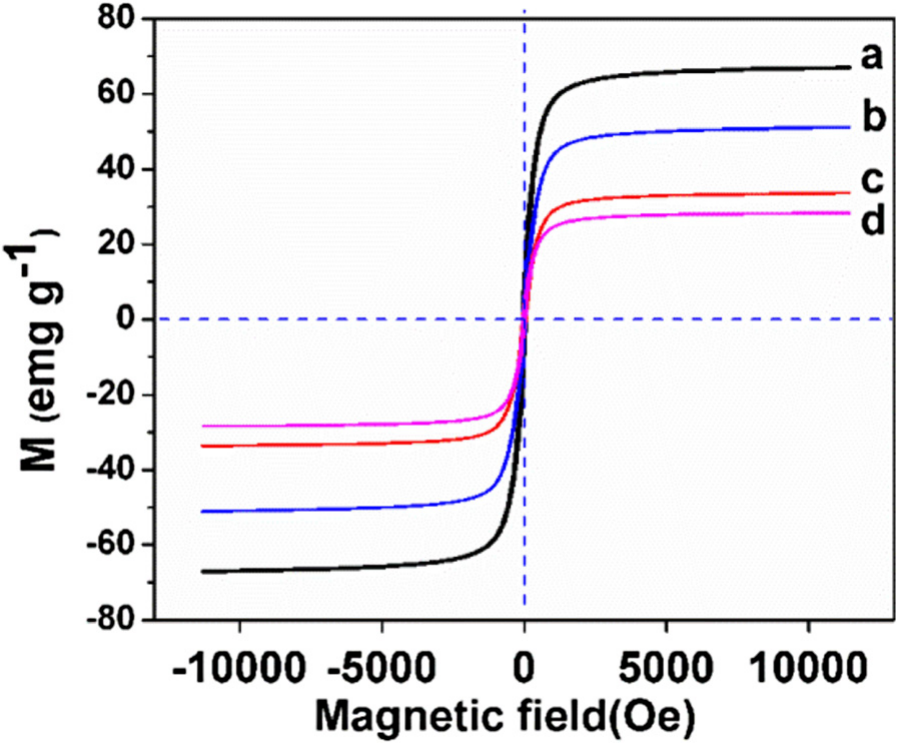
Room temperature (300 K) magnetic hysteresis loops of *a* Fe_3_O_4_ NPs, *b* Fe_3_O_4_@SiO_2_, *c* Fe_3_O_4_@SiO_2_-[Eu(DBM)_3_L_p_], *d* Fe_3_O_4_@SiO_2_-[Eu(DBM)_3_L_p_]@PEI nanocomposite

Magnetic characterization at 300 K with a VSM showed that the saturation magnetization values of Fe_3_O_4_NPs, Fe_3_O_4_@SiO_2_, Fe_3_O_4_@SiO_2_-[Eu(DBM)_3_L_p_], and the PEI-modified nanocomposite were 68.7, 51.0, 32.3, and 27.4 emu g^−1^ (Fig. 4), respectively, and the magnified hysteresis loops further confirmed the superparamagnetism of these NPs. Though the saturation magnetization of the Fe_3_O_4_@SiO_2_-[Eu(DBM)_3_L_p_]@PEI nanocomposite is less than the magnetite NPs as magnetic core, it may be believed to possess enough strong magnetic attraction for effectively magnetic targeting and separation.

**Fig. 5:**
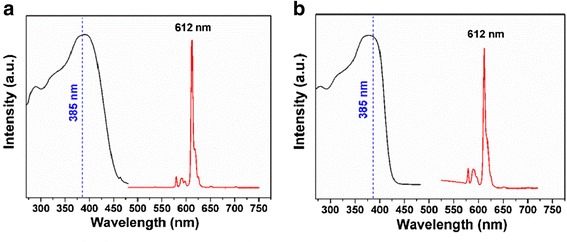
Excitation (*black line*, monitored at 612 nm) and emission (*red line*, monitored at 385 nm) spectra of the complex **a** Eu(DBM)_3_L_p_ and **b** the Fe_3_O_4_@SiO_2_-[Eu(DBM)_3_L_p_]@PEI nanocomposite

**Fig. 6:**
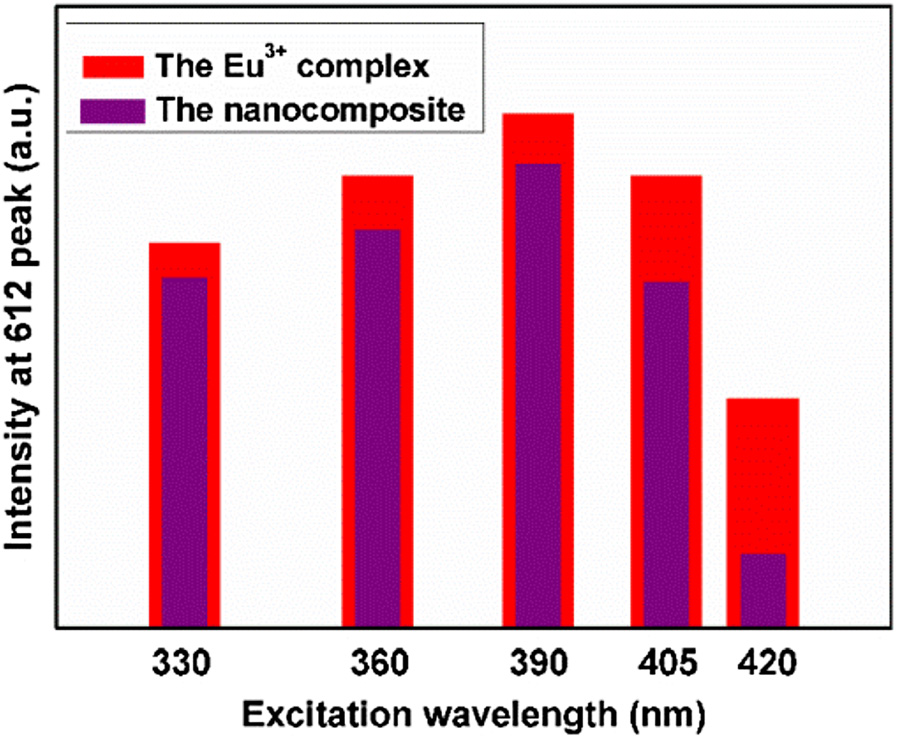
Comparison of the intensities of emission at 612 nm for the complex [Eu(DBM)_3_L_p_] with Fe_3_O_4_@SiO_2_-[Eu(DBM)_3_L_p_]@PEI nanocomposite at different excitation wavelengths

Figure 5 illustrates the corrected excitation (left) and emission (right) spectra of the isolated Eu^3+^ complex [Eu(DBM)_3_L_p_] and the Fe_3_O_4_@SiO_2_-[Eu(DBM)_3_L_p_]@PEI nanocomposite as solid at room temperature. The excitation spectra which were both obtained by monitoring at 612 nm exhibited a broad excitation band (BEB) between 250 and 450 nm. In the emission spectra, only characteristic emission of Eu(III) arising from the transition ^5^D_0_ → ^7^F_*J*_ (*J* = 0, 1, 2, 3, 4) was detected with the transition ^5^D_0_ → ^7^F_2_ (red emission) as the dominant group, which indicated that an efficient energy transfer from the ligands to Eu(III) could take place not only in the Eu(III) complex but also in the complex incorporated in the matrix [23]. Figure 6 shows the comparison of luminescence intensities of the Eu(III) complex [Eu(DBM)_3_L_p_] with the Fe_3_O_4_@SiO_2_-[Eu(DBM)_3_L_p_]@PEI nanocomposite at different excitation wavelengths from the UV to visible range (330, 360, 390, 405, and 420 nm). From these comparisons, one could note that both the precursor and the nanocomposite exhibited significant luminescent efficiency at different excitation wavelengths, which demonstrated the potential utility of this novel material in bioimaging [24].

The quantum yield (*Φ*) of the Fe_3_O_4_@SiO_2_-[Eu(DBM)_3_L_p_]@PEI nanocomposite was measured to be 2.70 % lower than that of the free Eu(DBM)_3_L_p_ (14 %), which might be due to the absorption of the matrix. The excited-state lifetime of the Fe_3_O_4_@SiO_2_-[Eu(DBM)_3_L_p_]@PEI NPs is 0.32 ms, which was close to that of the free Eu(DBM)_3_L_p_ (0.48 ms), indicating that the assembled nanocomposite was capable of eliminating background emission from a biological background for sensitive optical-imaging applications. The shortened lifetime might have given rise to the quenching of O-H oscillators on the matrix surfaces and the non-radiative dissipation of energy on the high-energy N-H vibrations from the modified PEI molecule, which also made the quantum yield of Fe_3_O_4_@SiO_2_-[Eu(DBM)_3_L_p_]@PEI nanocomposite be lower than that of Eu(DBM)_3_L_p_ [25].

**Fig. 7:**
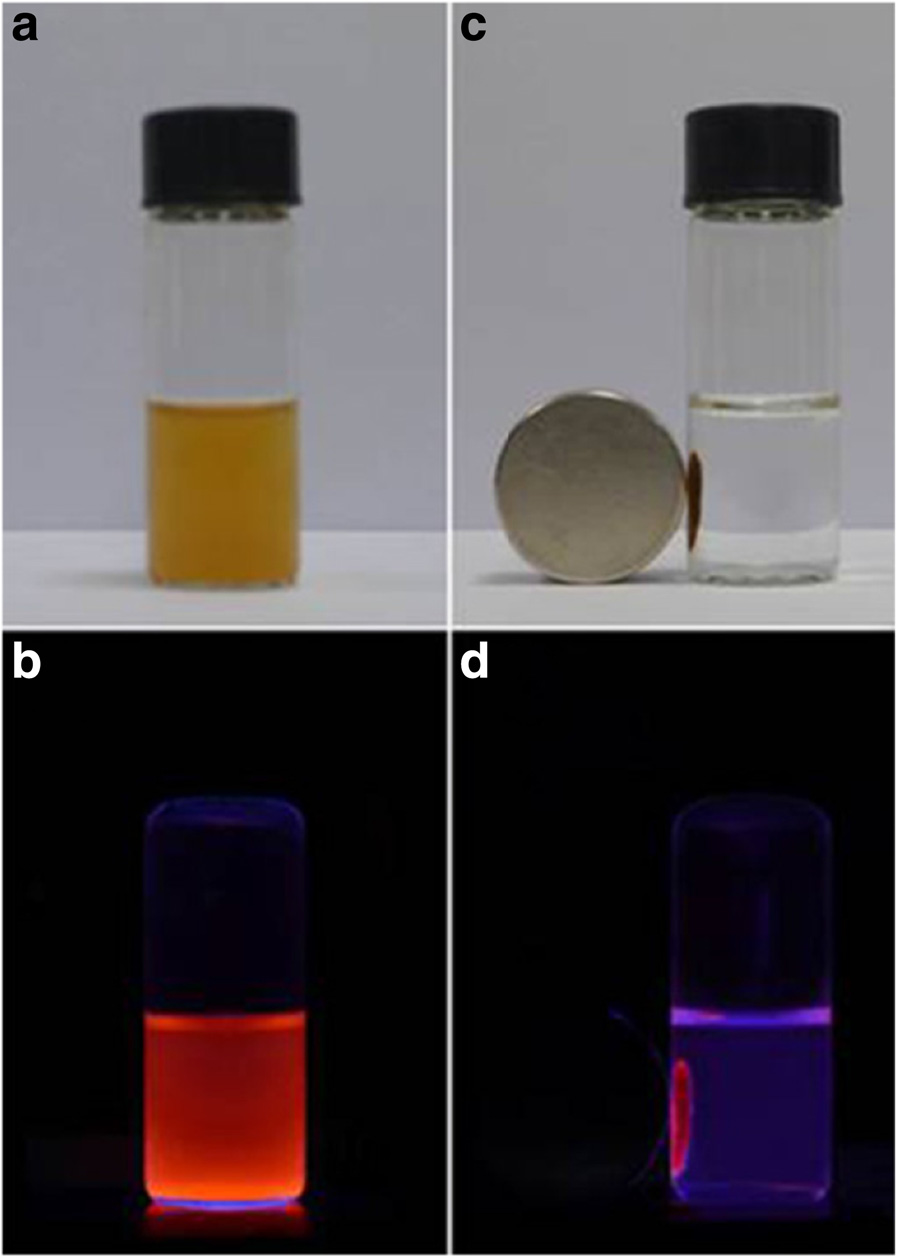
Photographs of the obtained bifunctional magnetic-optical nanoparticles before (**a**, **c**) and after (**b**, **d**) UV light irradiation (*λ* = 365 nm). **a**, **b** Aqueous suspension of the obtained bifunctional magnetic-luminescent nanoparticles. **c**, **d** After magnetic capture

Direct proof of magnetic-luminescent properties of the final product could be found in the following designed experiments. Upon UV light irradiation, the well-dispersed aqueous Fe_3_O_4_@SiO_2_-[Eu(DBM)_3_L_p_]@PEI nanocomposite emitted bright-red light originating from the characteristic emission of Eu(III) as shown in the digital photographs of Fig. 7b. When a handheld magnet was placed close to the glass vial, the nanocomposite particles were attracted to the magnet very quickly (Fig. 7c). Meanwhile, corresponding bright-red light emissions could be observed at these positions under UV light irradiation (Fig. 7d). After removal of the external magnet and sonication, the magnetic microspheres could be rapidly redispersed again. These results showed that the Fe_3_O_4_@SiO_2_-[Eu(DBM)_3_L_p_]@PEI nanocomposite possessed excellent magnetic responsiveness, luminescent property, and water solubility, which were important in terms of the practical manipulation.

The intense luminescent character of the Eu(III) complexes enables us to directly capture the Fe_3_O_4_@SiO_2_-[Eu(DBM)_3_L_p_]@PEI by using a fluorescence microscope. One of the advantages of using a fluorescence microscope as a tool is that the dispersive and luminescent properties at a micro level can be monitored. In the current study, observations using a fluorescence microscope were carried out on the Fe_3_O_4_@SiO_2_-[Eu(DBM)_3_L_p_]@PEI nanocomposite dispersed in PBS solution. When the nanocomposite was sonicated with a concentration of 0.1 wt.%, a stable dispersion was formed. Inside this dispersion, shinning red spots were observed as shown in Additional file 1: Figure S4, indicating the luminescent character of the composite. For comparison, Fe_3_O_4_@SiO_2_-[Eu(DBM)_3_L_p_] were dispersed in PBS solution at the same condition; however, great agglomeration could be seen from the photographs (Additional file 1: Figure S5). The most obvious reason for this phenomenon was that PEI modification significantly increased the water solubility of the nanocomposite.

## Conclusions

In summary, a simple and versatile strategy has been developed to transform Fe_3_O_4_ NPs into hydrophilic and biocompatible magnetic-luminescent dual-functional nanocomposites. The silica shells formed via the sol-gel method played significant roles in terms of trapping Fe_3_O_4_ NPs; the Eu(III) complex grafted made the NPs potential in a time-resolved imaging, and the PEI surface layer endowed the final material with hydrophilic and modifiable properties. The nanocomposite obtained was characterized by various techniques, and the results showed the desired properties as designed. Furthermore, this approach of functionalizing magnetic-luminescent nanocomposite via hydrogen-bonding method may be applied to fabricate other nanocomposites in order to obtain broader optical properties and potential applications.

## Additional File

Additional file 1: Figure S1. DLS results of the samples. (a) Eu(DBM)_3_L_p_ complex; (b) Fe_3_O_4_@SiO_2_; (c) Fe_3_O_4_@SiO_2_-[Eu(DBM)_3_L_p_] and (d) Fe_3_O_4_@SiO_2_-[Eu(DBM)_3_L_p_]@PEI. Figure S2. FTIR spectra and images obtained under a fluorescence microscope for Fe_3_O_4_@SiO_2_-[Eu(DBM)_3_Lp] nanocomposite]. Figure S3. a typical image obtained under a fluorescence microscope for Fe_3_O_4_@SiO_2_-[Eu(DBM)_3_L_p_]@PEI composite dispersed in PBS solution. Images a, b, c were taken at different regions of the same sample. Figure S4. A typical image obtained under a fluorescence microscope for Fe_3_O_4_@SiO_2_–[Eu(DBM)_3_L_p_] nanocomposite dispersed in PBS solution. Images a, b, and c were taken at different regions of the same sample. Figure S5. The PXRD analysis of the Fe_3_O_4_ NPs. (DOC 2084 kb)

## References

[1] Wang BD, Hai J, Wang Q, Li TR, Yang ZY (2011). Coupling of luminescent terbium complexes to Fe_3_O_4_ nanoparticles for imaging applications. Angew. Chem. Int. Ed..

[2] Chan CF, Tsang MK, Li HG, Lan RF, Chadbourne FL, Chan WL, Law GL, Cobb SL, Hao JH, Wong WT, Wong KL (2014). Bifunctional up-converting lanthanide nanoparticles for selective *in vitro* imaging and inhibition of cyclin D as anti-cancer agents. J. Mater. Chem. B.

[3] Koo CK, Wong KL, Man CWY, Tam HL, Tsao SW, Cheah KW, Lam MHW (2009). Two-photon plasma membrane imaging in live cells by an amphiphilic, water-soluble cyctometalated platinum(II) complex. Inorg. Chem..

[4] Lu D, Teng F, Liu YC, Lu LJ, Chen C, Lei JY, Wang LZ, Zhang JL (2014). Self-assembly of magnetically recoverable ratiometric Cu^2+^ fluorescent sensor and adsorbent. RSC Adv..

[5] Shen JH, Zhu YH, Yang XL, Zong J, Li CZ (2013). Multifunctional Fe_3_O_4_@Ag/SiO_2_/Au core-shell microspheres as a novel SERS-activity label via long-range plasmon coupling. Langmuir.

[6] Li JG, Jiang H, Yu ZQ, Xia HY, Zou G, Zhang QJ, Yu Y (2013). Multifunctional uniform core-shell Fe_3_O_4_@mSiO_2_ mesoporous nanoparticles for bimodal imaging and photothermal therapy. Chem. Asian J..

[7] Anders CB, Chess JJ, Wingett DG, Punnoose A (2015). Serum proteins enhance dispersion stability and influence the cytotoxicity and dosimetry of ZnO nanoparticles in suspension and adherent cancer cell models. Nanoscale Res. Lett..

[8] Comby S, Surender EM, Kotova O, Truman LK, Molloy JK, Gunnlaugsson T (2014). Lanthanide-functionalized nanoparticles as MRI and luminescent probes for sensing and/or imaging applications. Inorg. Chem..

[9] Yip YW, Wen H, Wong WT, Tanner PA, Wong KL (2012). Increased antenna effect of the lanthanide complexes by control of a number of terdentate N-donor pyridine ligands. Inorg. Chem..

[10] Armelao L, Dell’Amico DB, Bellucci L, Bottaro G, Labella L, Marchetti F, Samaritani S (2016). Smart grafting of lanthanides onto silica via N, N-dialkylcarbamato complexes. Inorg. Chem..

[11] Jin J, Yang F, Zhang FW, Hu WQ, Sun SB, Ma JT (2012). 2, 2'-(Phenylazanediyl) diacetic acid modified Fe_3_O_4_@PEI for selective removal of cadmium ions from blood. Nanoscale.

[12] Bae KH, Lee K, Kim C, Park TG (2011). Surface functionalized hollow manganese oxide nanoparticles for cancer targeted siRNA delivery and magnetic resonance imaging. Biomatertials..

[13] Date T, Sekine J, Matsuno H, Serizawa T (2011). Polymer-binding peptides for the noncovalent modification of polymer surfaces: effects of peptide density on the subsequent immobilization of functional proteins. ACS Appl. Mater. Interfaces.

[14] Knowles KR, Hanson CC, Fogel AL, Warhol B, Rider DA (2012). Layer-by-layer assembled multilayers of polyethylenimine-stabilized platinum nanoparticles and PEDOT:PSS as anodes for the methanol oxidation reaction. ACS Appl. Mater. Interfaces.

[15] Zucchi G, Murugesan V, Tondelier D, Aldakov D, Jeon T, Yang F, Thuéry P, Ephritikhine M, Geffroy B (2011). Solution, solid state, and film properties of a structurally characterized highly luminescent molecular europium plastic material excitable with visible light. Inorg. Chem..

[16] Xuan SH, Wang YXJ, Yu JC, Leung KCF (2009). Tuning the grain size and particle size of superparamagnetic Fe_3_O_4_ microparticles. Chem. Mater..

[17] Sikora A, Shard AG, Minelli C (2016). Size and ζ-potential measurement of silica nanoparticles in serum using tunable resistive pulse sensing. Langmuir.

[18] Baek SY, Na K (2013). A nano complex of hydrophilic phthalocyanine and polyethylenimine for improved cellular internalization efficiency and phototoxicity. Colloids Surf. B Biointerfaces.

[19] Akbarzadeh A, Samiei M, Davaran S (2012). Magnetic nanoparticles: preparation, physical properties, and applications in biomedicine. Nanoscale Res. Lett..

[20] Lei BF, Li B, Zhang HR, Zhang LM, Li WL (2007). Synthesis, characterization, and oxygen sensing properties of functionalized mesoporous SBA-15 and MCM-41 with a covalently linked ruthenium(II) complex. J. Phys. Chem. C.

[21] Peng CQ, Thio YS, Gerhardt RA (2010). Magnetic nanoparticles: preparation, physical properties, and applications in biomedicine. J. Phys. Chem. C.

[22] Liu QY, Jia QY, Zhu JQ, Shao Q, Fan JF, Wang DM, Yin YS (2014). Highly ordered arrangement of meso-tetrakis(4-aminophenyl)porphyrin in self-assembled nanoaggregates via hydrogen bonding. Chinese Chem. Lett..

[23] Divya V, Reddy MLP (2013). Visible-light excited red emitting luminescent nanocomposites derived from Eu^3+^-phenanthrene-based fluorinated β-diketonate complexes and multi-walled carbon nanotubes. J. Mater. Chem. C.

[24] Reddy MLP, Divya V, Pavithran R (2013). Visible-light sensitized luminescent europium(III)-β-diketonate complexes: bioprobes for cellular imaging. Dalton Trans..

[25] Xu J, Sun Z, Jia L, Li B, Zhao L, Liu X, Ma Y, Tian H, Wang Q, Liu W, Tang Y (2011). Visible light sensitized attapulgite-based lanthanide composites: microstructure, photophysical behaviour and biological application. Dalton Trans..

