# Supplementary material for: Lanthanide-Functionalized Hydrophilic Magnetic Hybrid Nanoparticles: Assembly, Magnetic Behaviour, and Photophysical Properties
Source: Nanoscale Res Lett. 2016 May 31;11:273. doi: 10.1186/s11671-016-1497-3 (PMC4887399; doi:10.1186/s11671-016-1497-3)
Supplement: Additional file 1: — Figure S1. DLS results of the samples. (a) Eu(DBM)3Lp complex; (b) Fe3O4@SiO2; (c) Fe3O4@SiO2-[Eu(DBM)3Lp] and (d) Fe3O4@SiO2-[Eu(DBM)3Lp]@PEI. Figure S2. FTIR spectra and images obtained under a fluorescence microscope for Fe3O4@SiO2-[Eu(DBM)3Lp] nanocomposite]. Figure S3. a typical image obtained under a fluorescence microscope for Fe3O4@SiO2-[Eu(DBM)3Lp]@PEI composite dispersed in PBS solution. Images a, b, c were taken at different regions of the same sample. Figure S4. A typical image obtained under a fluorescence microscope for Fe3O4@SiO2–[Eu(DBM)3Lp] nanocomposite dispersed in PBS solution. Images a, b, and c were taken at different regions of the same sample. Figure S5. The PXRD analysis of the Fe3O4 NPs. (DOC 2084 kb) [file 11671_2016_1497_MOESM1_ESM.doc]

**Supporting information**

Lanthanide-functionalized hydrophilic magnetic hybrid nanoparticles: assembly, magnetic behaviour and photophysical properties

Shuai Han^1＊^, Yu Tang^2^, Haijun Guo^1^, Shenjun Qin^1^**,** Jiang Wu^2^

1. *College of Science, Hebei University of Engineering, Handan 056000, PR China*
2. *Key Laboratory of Nonferrous Metal Chemistry and Resources Utilization of Gansu Province, State Key Laboratory of Applied Organic Chemistry and College of Chemistry and Chemical Engineering, Lanzhou University, Lanzhou, 730000 (P. R. China)*


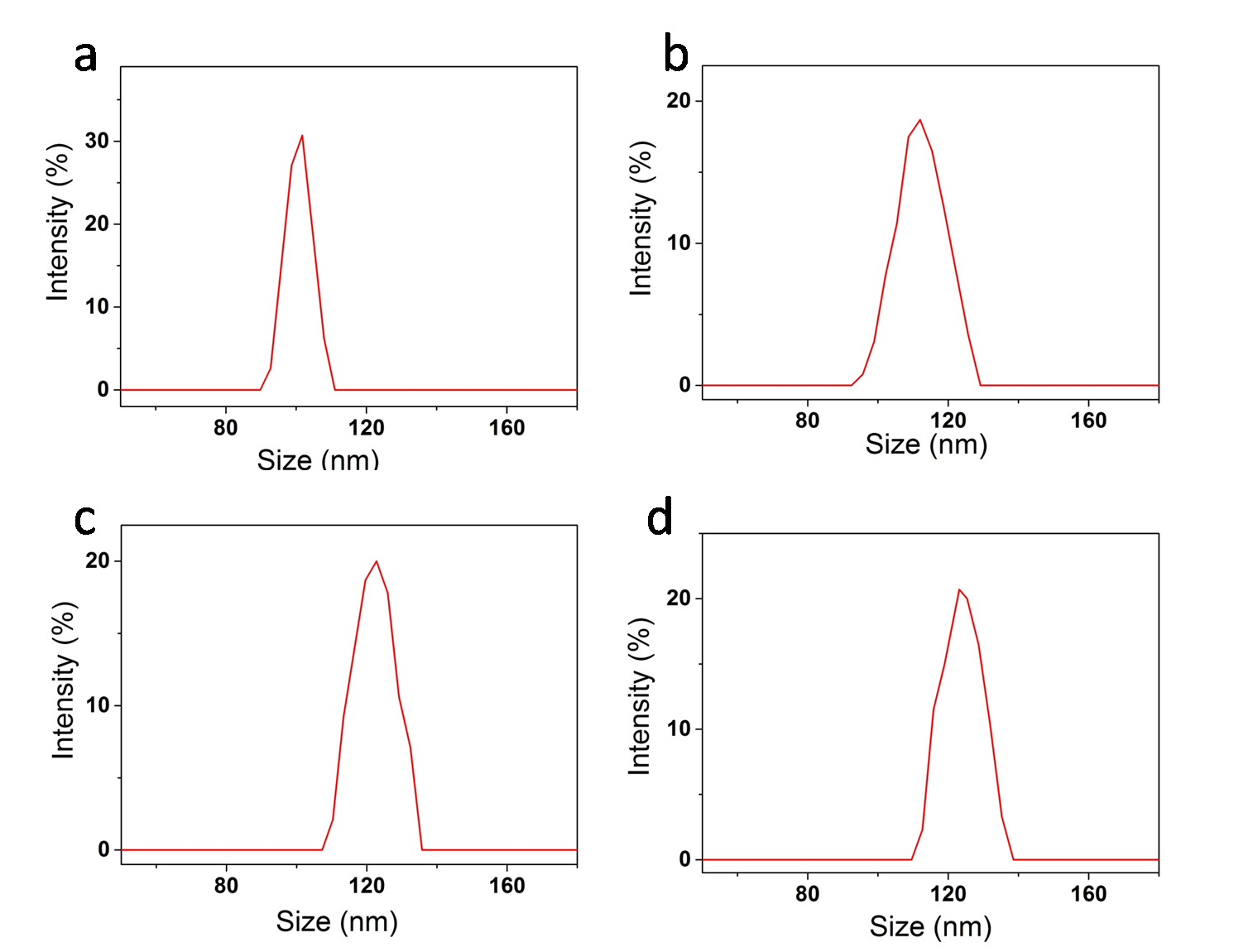


**Fig. S1**  DLS results of the samples. (a) Eu(DBM)_3_L_p_ complex; (b) Fe_3_O_4_@SiO_2_; (c) Fe_3_O_4_@SiO_2_-[Eu(DBM)_3_L_p_] and (d) Fe_3_O_4_@SiO_2_-[Eu(DBM)_3_L_p_]@PEI.


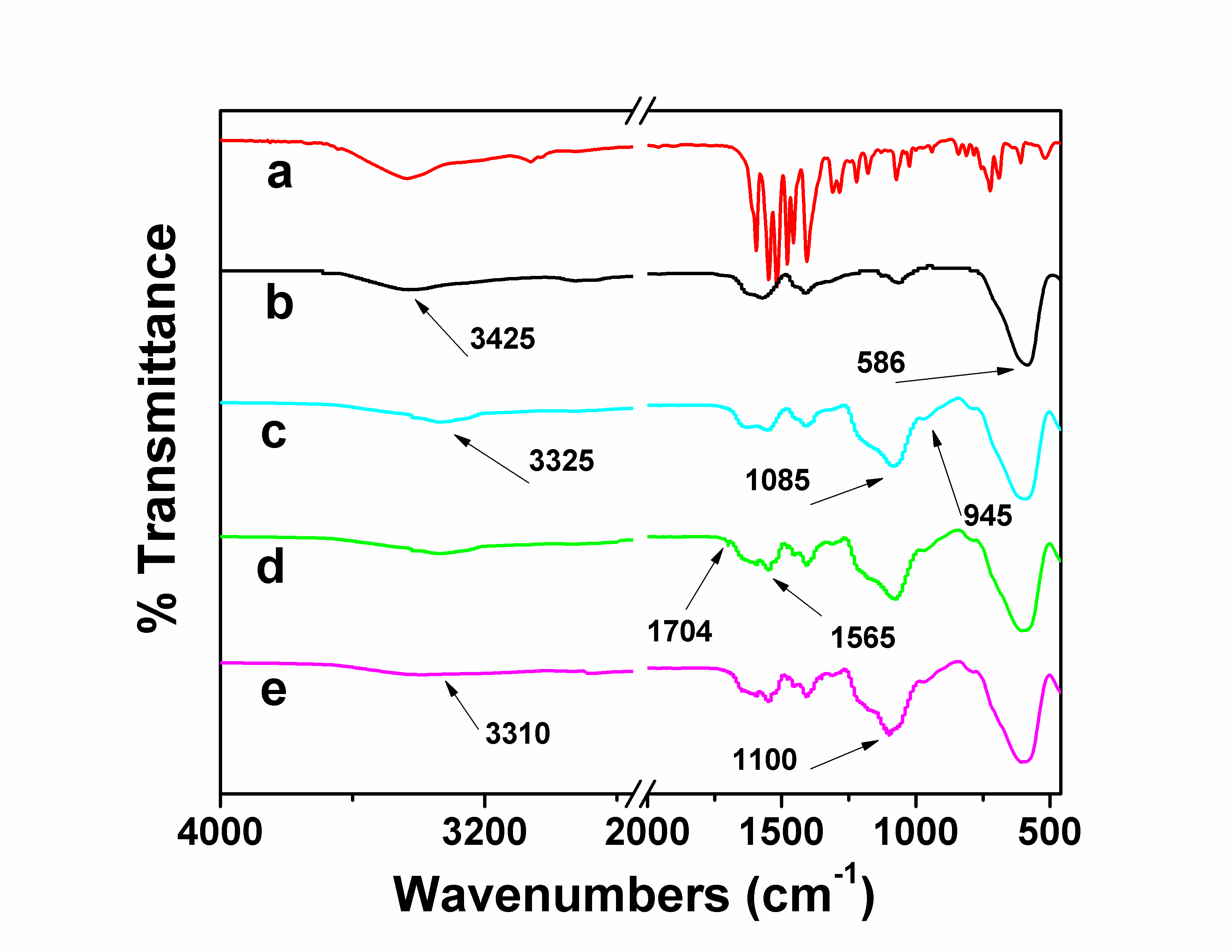


**Fig. S2**  FTIR spectra of of (a) Eu(DBM)_3_L_p_ complex, (b) Fe_3_O_4_ NPs, (c) Fe_3_O_4_@SiO_2_, (d) Fe_3_O_4_@SiO_2_–[Eu(DBM)_3_L_p_], and (e) Fe_3_O_4_@SiO_2_–[Eu-(DBM)_3_L_p_]@PEI.


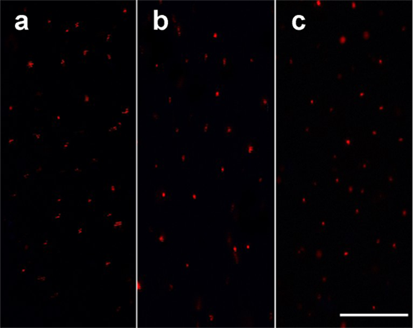
**Fig. S3** a typical image obtained under a fluorescence microscope for Fe_3_O_4_@SiO_2_-[Eu(DBM)_3_L_p_]@PEI composite dispersed in PBS solution. Images a, b, c were taken at different regions of the same sample. The scale bar is 10 µm


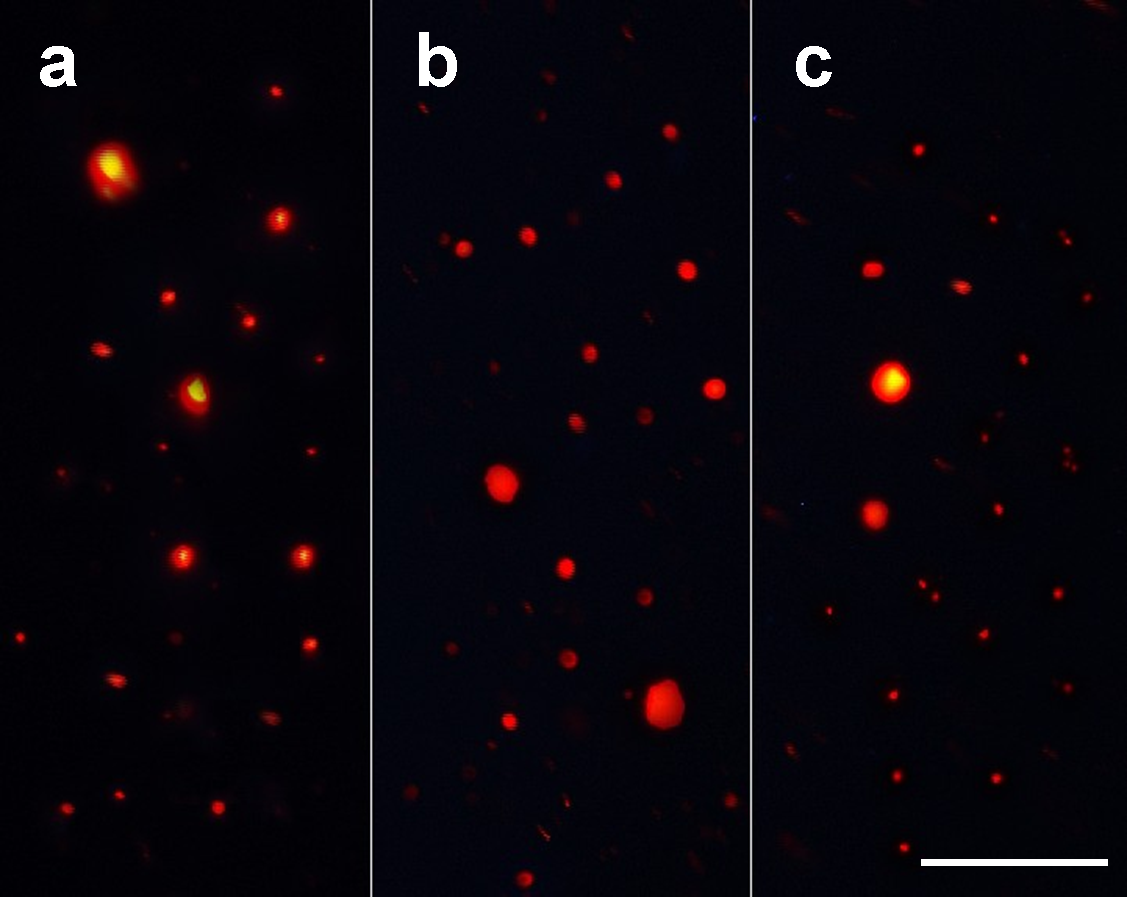


**Fig. S4** A typical image obtained under a fluorescence microscope for Fe_3_O_4_@SiO_2_–[Eu(DBM)_3_L_p_] nanocomposite dispersed in PBS solution. Images a, b, and c were taken at different regions of the same sample. The scale bar is 10 µm.


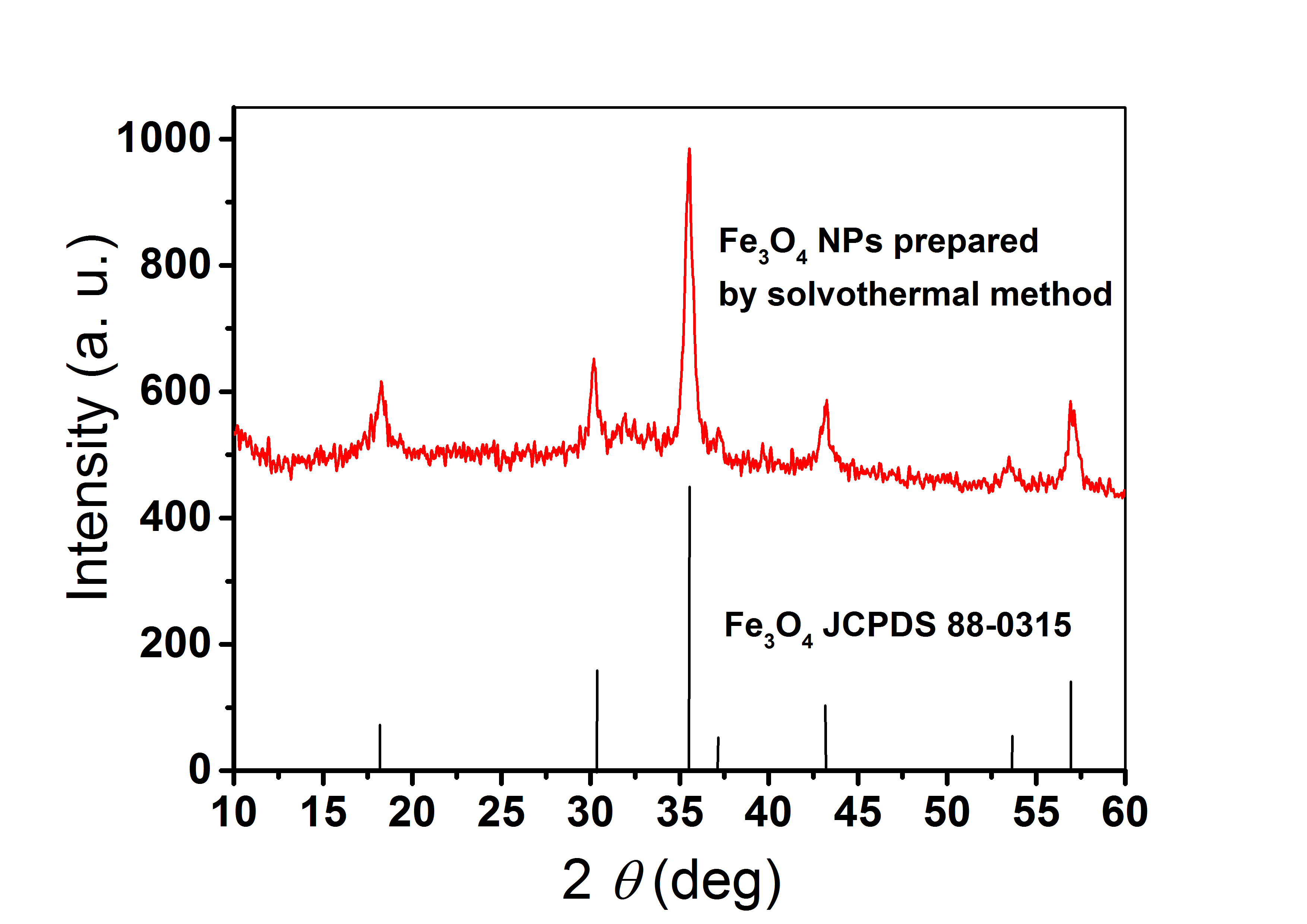


Fig. S5 The PXRD analysis of the Fe_3_O_4_ NPs
